# Attenuation of oxidative stress and chromosomal aberrations in cultured macrophages and pulmonary cells following self-sustained high temperature synthesis of asbestos

**DOI:** 10.1038/s41598-020-65620-x

**Published:** 2020-05-22

**Authors:** Sebastiano La Maestra, Rosanna T. Micale, Maurizio Ferretti, Alberto Izzotti, Laura Gaggero

**Affiliations:** 1 Department of Health Sciences, University of Genoa, via A. Pastore, 1, 16132 Genoa, Italy; 2 Department of Chemistry and Industrial Chemistry, University of Genoa, Via Dodecaneso 31, 16146 Genoa, Italy; 3 Mutagenesis and Cancer Prevention Unit, IRCCS Ospedale Policlinico San Martino, Largo R. Benzi, 10, 16132 Genoa, Italy; 4 Department of Earth, Environment and Life Sciences, University of Genoa, Corso Europa 26, 16132 Genoa, Italy

**Keywords:** Environmental sciences, Health care, Risk factors

## Abstract

Inhalation of asbestos fibres can cause lung and pleural diseases in humans and constitutes a severe public health threat worldwide. The aim of the present study was to assess the biological effects induced in both pulmonary cells (A549) and monocyte/macrophage (RAW 264.7) cell lines by combustion slags obtained from asbestos through a self-sustained high-temperature synthesis (SHS) reaction. The SHS reaction involves rapid thermal treatment and displays great ability to neutralise asbestos. Cytotoxicity, redox status imbalance, lipid peroxide production, DNA strand breaks (comet assay) and chromosomal aberrations (cytokinesis block micronucleus test) were evaluated in cells exposed either to untreated asbestos fibres or to grinded SHS-generated slags of different granulometry, tested in cultured cells at varying doses and for varying exposure times. Our results show that asbestos fibres cause redox status imbalance, especially in monocyte/macrophage cell lines. Moreover, they promote lipid peroxidation and trigger genomic alterations. When the cells were exposed to slag powders, which are the products of SHS asbestos treatment, generation of lipid peroxides and induction of DNA strand breaks still persisted, due to the high content in iron and other metals detected in these samples. However, there was an attenuation of redox status imbalance and an absence of chromosomal aberrations, which probably reflects the loss of the asbestos fibrous structure following SHS reaction, as demonstrated by electron microscopy analyses. In conclusions, SHS-treated asbestos wastes can potentially have deleterious health effects due to the oxidative stress induced by inhaled powders but they loose the asbestos ability to induce chromosomal alterations.

## Introduction

Asbestos is a generic name covering a group of hydrated fibrous mineral silicates. It has widely been used for many years in heavy industries, primarily in construction materials and in many friction products, because it is affordable and durable. Two types of asbestos can be distinguished on the basis of their capacity to split into long, slender, resilient, tough fibres, and ultimately fibrils (single fibres), including serpentine (chrysotile) and amphibole (crocidolite, amosite, and tremolite), the latter being more dangerous than the former. Owing to their aerodynamic properties, fine asbestos fibres can easily be inhaled and can cause several respiratory alterations, including a potentially serious lung fibrosis called asbestosis. Exposure to asbestos can also cause both extrapleural and pleural mesothelioma as well as cancer of the lungs, larynx and ovaries^1^.

A heavy exposure to asbestos occurred in workers employed in the shipbuilding industry, asbestos-removal workers, mine workers and even in residents in geographical areas that were formerly exposed to asbestos^2^. Banning the use of asbestos and appropriately managing contaminated wastes are the most effective strategies for safeguarding both workers and subjects exposed non-professionally to asbestos-related risks. In fact, exposure to asbestos constitutes a significant cancer risk factor not only in asbestos workers but an indirect exposure also poses a threat to family members^3^. Although the number of industries that still use asbestos is decreasing, the workers with the highest risks today are likely to be those subject to incidental exposures, for example construction workers and tunnel excavation workers. However, awareness of this issue has increased, and advanced preventive monitoring in the workplace is currently carried out^4^.

Asbestos is a bio-persistent mineral known to trigger processes of carcinogenicity owing to its physical/chemical properties^5^. Nevertheless, asbestos use in various industrial processes is unlikely to be abandoned soon. Thus, prevention programmes and policy measures must be followed by changes in the management of existing structures, devices and equipment, and removal operations^6^.

The World Health Organisation (WHO) and the International Labour Organisation (ILO) have stated that the most effective way to eliminate asbestos-related diseases is to forbid the use of the various forms of asbestos^7,8^. Despite the ban in 55 countries, asbestos is still widely used. Recent analysis estimates asbestos consumption to be 2,030,000 tons annually, with about 90% of the world’s supply currently coming from Russia, China, Brazil and Kazakhstan^9,10^. In Italy, the quantity of asbestos-containing waste (ACW) is thought to be as much as 30 million tons, i.e., 18 million m^3^, about half of which is pure asbestos^11^, and the situation in other European countries is similar.

Asbestos can contaminate superficial and deep waters and the surrounding environment if dispersed; to tackle this threat, the best solution would be to implement inertisation processes. An associated problem is the high cost of disposal of ACW, which is in the order of 1.50 Euro/kg^12^. It is therefore essential to avoid, or to minimize as far as possible, the amount of waste created by adopting the most efficient production techniques and by recycling. On firing at >1000 °C, both serpentine and amphibole types, which are hydrated silicates, are transformed into anhydrous Mg–Fe silicates, such as olivine^13^. The mineral obtained can be reutilised as a secondary raw material or disposed in sites for inert waste, such as low-impact landfilling.

The Italian legislation strictly requires that the final status of waste mixtures from combusted asbestos should be assessed by means of Scanning Electron Microscope coupled with Energy Dispersive Spectrometry (SEM-EDS) and XR Powder Diffraction (XRPD) analysis, in order to ascertain that the minerals have been broken down both physically and chemically and that no fibres remain. In Europe, the 2008/98/EC Directive on waste encourages reduction, recycling and recovery before disposal. Landfilling requires low energy and is fast and does not destroy fibres. Several patents are based on conventional thermal treatments, which however are energivore and long lasting. Therefore, the assessment of effective neutralisation of the ACW by a fast and low energy treatment could support an environment sustainable solution for such a hazardous waste.

To neutralise asbestos, a method of thermal treatment based on combustion synthesis, usually referred to as self-sustained high-temperature synthesis (SHS), was developed from the laboratory scale up to an industrial scale^14^, as shown in the Life FIBERS Project, LIFE12 ENV IT 000295, Fibers Innovative Burning and Reuse by SHS (http://www.fibers-life.eu). The ability of the fast combustion reaction to destroy the fibrous structure of asbestos is attained at high temperature (1200 °C), resulting in the generation of a slag that contains, among other phases, olivine, a non-fibrous silicate. This method efficiently addressed the passivation of natural asbestos fibres and asbestos-bearing artificial materials. The attainment of zero-fibre content is verified through DTA-TG thermal analyses (Laura Gaggero, Maurizio Ferretti & Federico Locardi, Assessment of post treatment acceptance for asbestos-containing waste by differential thermal and thermo-gravimetric analysis (DTA-TG), manuscript in preparation).

Unlike conventional thermal treatments, the SHS process requires a short reaction time and low activation energy, thereby yielding savings in both time and costs. The validation of treated waste products enables them to be re-used as secondary raw material, such as abrasive or refractory materials, thereby giving them a “second life”^14^. The final products of neutralisation are granular silicates and oxides (e.g., granular forsterite), which may be reused as secondary abrasive, refractory, or ceramic raw materials, or, at worst, as aggregates. Gaggero and Ferretti^13^ carried out post-treatment physical and chemical characterization of combusted pellets by means of optical microscopy, XRPD and SEM coupled with energy-dispersive X-ray spectroscopy (EDS) microanalysis.

In the present study, we subjected asbestos to SHS treatment and evaluated the effects of such a thermal process on morphological appearance and chemical composition of the treated slags in comparison with untreated asbestos. In addition, we examined the biological effects of powders generated by the comminution of treated slags, using untreated chrysotile fibres as positive controls. To this purpose, samples of different granulometry were tested at varying doses and for varying exposure times in both A549 cells, a human alveolar basal epithelial cell line derived from lung carcinoma, and RAW 264.7 cells, a mouse monocyte/macrophage cell line. The investigated end-points included assessment of the redox status, generation of lipid peroxides, DNA damage, and chromosomal aberrations.

## Results

### Ultrastructural appearance

**Figure 1:**
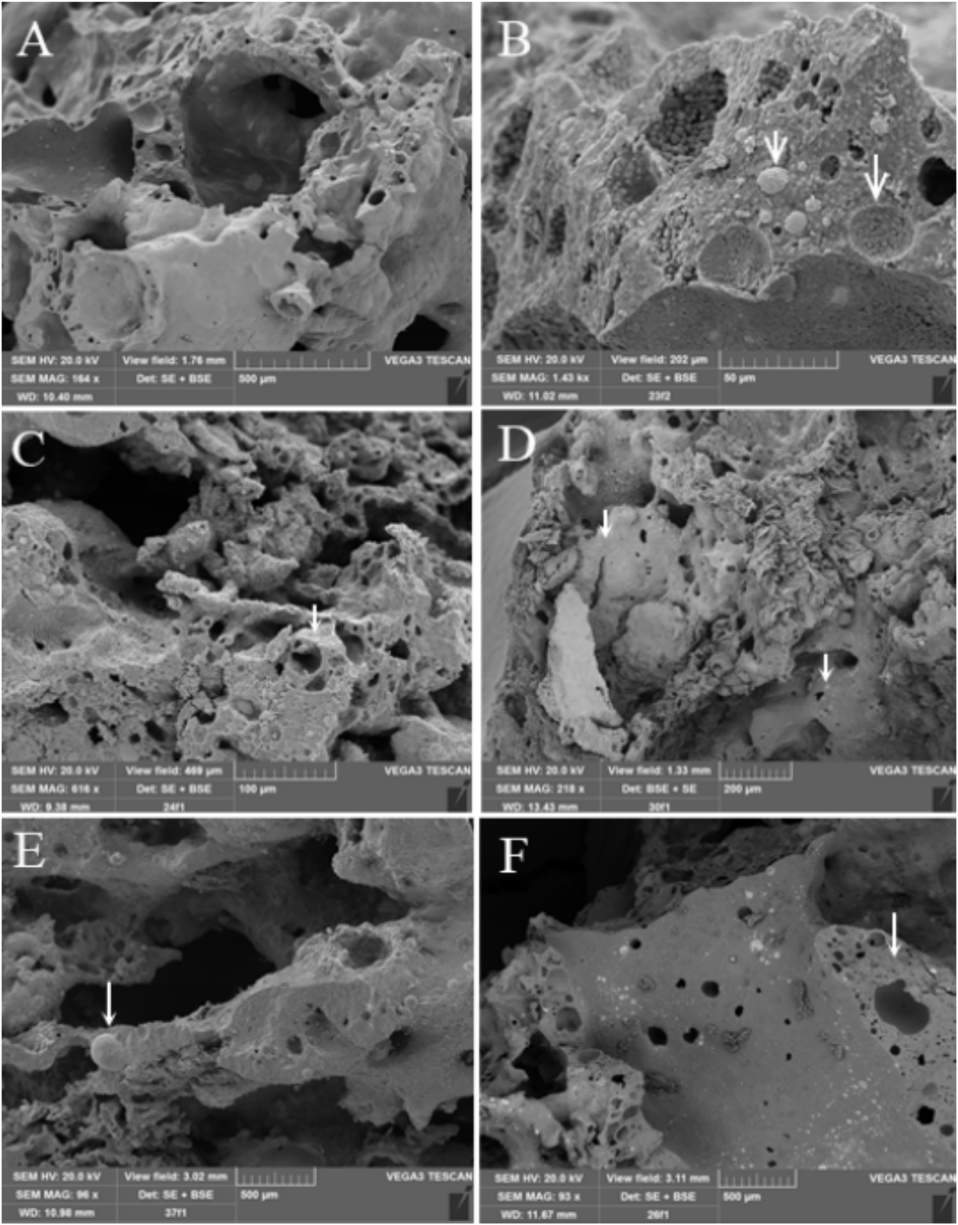
SEM microphotographs of SHS-treated pellets. (**A**) Scoriaceous texture. (**B**) Detail of A. Granular forsterite and wüstite pave the bubble cavities and globular metallic iron drops, suggesting that the T_melting_ of iron was attained. (**C**) Blocky, vesicular texture. (**D**) Scoriaceous texture showing an outer silicate shell (dark grey), including an inner metallic layer (light grey, arrow on the left). The arrow on the right indicates the inter-growth of globular metallic Fe within olivine. (**E**) Large globular metallic iron drop. (**F**) Scoriaceous texture of wüstite + forsterite, including irregularly scattered globular iron drops.

Figure 1A-F show examples of scanning electron microscopic (SEM) analyses of asbestos fibres after SHS treatment. The asbestos fibrous structure completely disappeared and the breakdown of chrysotile gave rise to newly formed olivine, a magnesium iron silicate, with the appearance of forsterite, a magnesium-rich end-member of olivine, and wüstite, a mineral form of iron(II) oxide. The volatile substances released from chrysotile or embedded calcite as breakdown products induced a swirling vesicular texture.

**Figure 2:**
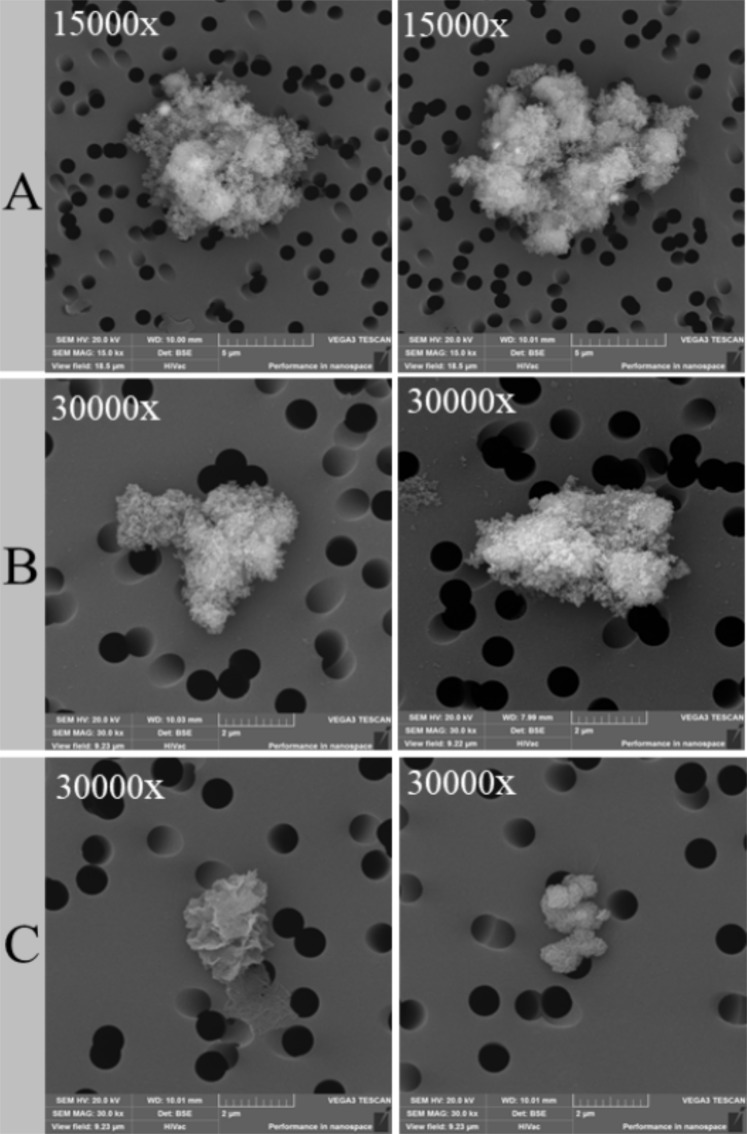
SEM microphotographs of the three grain-size fractions (<10 μm, <3 μm and <2 μm), referred to as (**A**–**C**), respectively. Powder particles were obtained from SHS-treated waste to be tested in A549 and Raw 267.4 cell lines.

Figure 2 reports representative examples of SEM microphotographs showing the morphology and size of the three SHS-treated waste fractions to be tested in cultured cells, having grain sizes of <10 μm (A), <3 μm (B) and <2 μm (C).

### Chemical composition

Table 1 reports the bulk composition of 5 SHS-treated slags, as assessed by Fusion-Inductively Coupled Plasma (FUS-ICP). The main analytes detected were Fe_2_O_3_ (T), Al_2_O_3_, SiO_2_, and MgO. Excepting CaO, all the other analytes contributed for less than 1% to the composition of the analysed samples.

**Table 1:** Representative bulk composition of 5 SHS-treated slag samples analyzed by Fusion Inductively Coupled Plasma (FUS-ICP).

Analyte	Weight (%)					
	Detection Limit	#1	#2	#3	#4	#5
SiO_2_	0.01	12.94	12.76	16.65	9.24	14.05
Al_2_O_3_	0.01	39.36	32.69	26.05	31.60	37.02
Fe_2_O_3_(T)	0.01	35.58	32.14	42.82	49.38	38.24
MnO	0.001	0.14	0.23	0.15	0.18	0.16
MgO	0.01	10.32	20.70	10.64	8.04	3.03
CaO	0.01	0.58	0.80	1.10	0.44	5.69
Na_2_O	0.01	0.07	0.04	0.12	0.02	0.24
K_2_O	0.01	0.05	0.01	0.11	<0.01	0.20
TiO_2_	0.001	0.11	0.05	0.08	0.03	0.08
P_2_O_5_	0.01	0.04	0.02	0.03	0.03	0.02
Loss on Ignition (LOI)		1.03	1.50	2.00	1.70	1.65
Total		100.22	100.95	99.76	100.66	100.38

### Viability of cells

Table 2 shows the viability of Raw 264.7 cells and A549 cells treated with varying concentrations (0.33, 1.00, 3.33, 7.58, and 15.62 µg/cm^2^ of cell culture surface) of powder particles of three diameters (<10 μm, <3 μm, and <2 μm) for different time periods (24, 48, and 72 h).Cell viability differed between the two cell lines. In fact, survival was higher in RAW 264.7 cells than in A549 cells, irrespective of the diameter of the particles. RAW 264.7 cells treated with powders of diameters <2 μm (C) showed a statistically significant cytotoxicity at the highest concentration tested (15.62 µg/cm^2^, C5). A noteworthy effect was the increase in the number of RAW 264.7 cells after 72 h, probably due to a mitogenic response induced by non-self materials in the growing medium^15,16^. A slight toxic effect was detected in A549 cells treated with the highest concentration of the particles with diameters <10 µm (A4–A5) after 48 and 72 h. The increase in toxicity in A549 cells treated with particles with diameters <3 µm (B1–B5) and <2 µm (C1–C5) was inversely related to the diameter of the particles and directly related to the contact time. Asbestos reduced the viability of both cell lines at the highest concentrations tested and at the longest exposure times.

**Table 2:** Viability of A549 cells and Raw 264.7 cells exposed for varying time periods either to asbestos or to SHS-treated slags of different granulometry (see Fig. 2).

Tested material	Amount (µg/cm^2^)	Raw 264.7 cells			A549 cells		
		24 h	48 h	72 h	24 h	48 h	72 h
Asbestos	0.33 (D1)	114.8 ± 2.10	116.1 ± 7.98^a^	122.7 ± 9.85^b^	98.5 ± 2.30	119.5 ± 1.30^c^	82.6 ± 5.57^b^
	1.00 (D2)	106.1 ± 1.76	104.1 ± 7.60	108.0 ± 6.30^a^	99.5 ± 3.30	115.8 ± 5.60^c^	88.4 ± 6.53^a^
	3.33 (D3)	87.6 ± 2.21^b^	72.6 ± 8.88^b^	50.0 ± 4.11^c^	97.2 ± 3.10	114.7 ± 4.60^c^	89.6 ± 8.30^a^
	7.58 (D4)	83.9 ± 1.29^b^	59.1 ± 8.99^c^	45.5 ± 7.09^c^	82.5 ± 1.50^a^	103.2 ± 2.60	88.2 ± 6.59
	15.62 (D5)	87.4 ± 1.69^b^	70.6 ± 2.60^c^	60.3 ± 3.08^c^	79.0 ± 0.70^a^	57.1 ± 4.60^c^	54.9 ± 5.60^c^
Powder <10 µm	0.33 (A1)	96.8 ± 0.60	95.8 ± 1.70	99.1 ± 7.32	113.1 ± 2.10	104.5 ± 8.70	92.5 ± 1.20^b^
	1.00 (A2)	98.6 ± 0.90	102.7 ± 4.51	105.5 ± 8.94	119.9 ± 3.60	105.2 ± 6.30	94.5 ± 8.40^b^
	3.33 (A3)	98.6 ± 1.40	103.0 ± 6.20	116.4 ± 8.81^b^	111.3 ± 1.60	103.3 ± 5.30	87.1 ± 9.40^b^
	7.58 (A4)	101.2 ± 0.80	103.0 ± 3.40	120.9 ± 9.62^c^	98.6 ± 2.40	81.2 ± 2.90^b^	86.5 ± 6.08^b^
	15.62 (A5)	100.5 ± 1.80	122.2 ± 4.23^c^	113.3 ± 6.70^b^	93.8 ± 0.90	91.5 ± 3.00^a^	93.7 ± 6.20
Powder <3 µm	0.33 (B1)	98.1 ± 2.11	83.8 ± 4.50	118.2 ± 3.20^b^	106.2 ± 1.10	94.8 ± 4.50	77.6 ± 6.80^b^
	1.00 (B2)	97.8 ± 1.25	89.8 ± 6.32	124.5 ± 4.00^c^	104.2 ± 1.60	97.2 ± 4.80	70.4 ± 3.70^c^
	3.33 (B3)	98.3 ± 2.20	94.8 ± 3.84	126.4 ± 8.09^c^	113.0 ± 2.90^a^	92.3 ± 4.10^b^	71.3 ± 6.30^c^
	7.58 (B4)	91.3 ± 1.28	110.5 ± 7.20^a^	103.4 ± 6.33	113.8 ± 2.20^b^	84.1 ± 2.50^b^	69.5 ± 7.50^c^
	15.62 (B5)	101.4 ± 1.73	113.6 ± 2.30^b^	117.8 ± 3.91^c^	93.8 ± 0.50^a^	88.8 ± 1.00^b^	80.7 ± 4.50^b^
Powder <2 µm	0.33 (C1)	96.3 ± 0.80	116.1 ± 7.00^a^	108.0 ± 11.12	103.8 ± 2.90^a^	114.7 ± 3.80^c^	80.2 ± 5.50^b^
	1.00 (C2)	100.6 ± 1.82	122.7 ± 7.81^b^	103.0 ± 9.02	89.2 ± 2.30^b^	111.8 ± 5.10^a^	76.9 ± 2.50^a^
	3.33 (C3)	99.6 ± 1.00	119.8 ± 3.33^b^	108.0 ± 9.89	90.5 ± 2.50	106.9 ± 5.50^b^	67.8 ± 12.28^b^
	7.58 (C4)	100.4 ± 0.98	103.8 ± 6.11	112.5 ± 9.50^a^	86.5 ± 1.40	109.3 ± 8.40^a^	65.8 ± 13.10^c^
	15.62 (C5)	85.7 ± 1.71^a^	97.7 ± 3.34	107.3 ± 2.30^a^	93.8 ± 0.80	64.8 ± 4.80^b^	62.6 ± 3.30^c^

The results are means ± SD of 8 replicates and are expressed as % of viability as compared with untreated controls of the corresponding cell lines.

Statistical analysis: ^a^P <0.05, ^b^P <0.01, and ^c^P <0.001, as compared with untreated controls.

### Redox status

**Figure 3:**
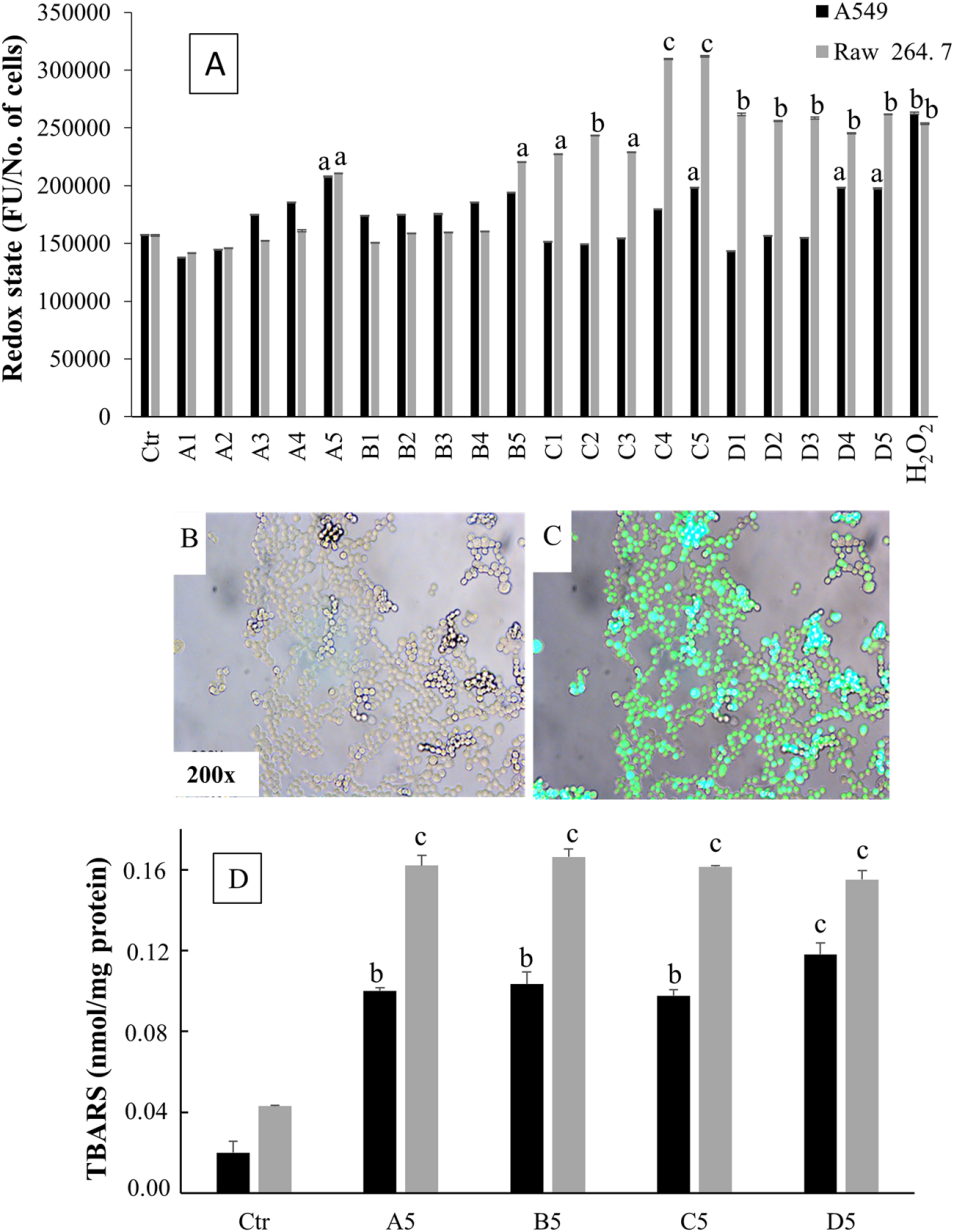
(**A**) Redox status assessed by means of DCF staining in A549 and Raw 267.4 cells. The redox status, expressed as intensity of DCF fluorescence, was evaluated in the two cell lines, either untreated (Ctr) or exposed for 3 h to varying concentrations either of slag powders with varying granulometry (A1–A5, B1–B5, C1–C5) or of untreated asbestos (D1–D5). H_2_O_2_ (100 µM) was tested as a positive control. The results are expressed as the average of fluorescence (FU)/number of cells and are the means ± SD of three independent experiments. Statistical analysis: ^a^*P* < 0.05, ^b^*P* < 0.01, and ^c^*P* < 0.001 *vs*. control. (**B**,**C**) Representative microphotographs showing the effect of slag powders in Raw 267.4 cell lines, either unstained (**B**) or loaded with DCFH-DA (**C). **(**D**) TBARS levels, expressed as nmol/mg protein, in A549 and Raw 267.4 cells after contact for 12 h with the highest concentrations either of slag powders (15.62 µg/cm^2^) of different granulometries (**A**–**C**) or of asbestos. (**D**) The columns report the means + SD of triplicate analyses. Statistical analysis: ^b^*P* < 0.01 and ^c^*P* < 0.001 *vs*. controls.

The redox status, expressed as intensity of DCF fluorescence, was evaluated in the two cell lines, either untreated (Ctr) or exposed for 3 h to varying concentrations either of slag powders with varying granulometry (A1–A5, B1–B5, C1–C5) or of untreated asbestos (D1–D5). H_2_O_2_ was tested as a positive control (Fig. 3A**)**. In general, RAW 264.7 cells were more susceptible than A549 cells to redox alterations induced either by slag powder particles or by untreated asbestos. The most intense fluorescence was observed in RAW 264.7 cells exposed to the powder particles of smallest granulometry.

The internalization of DCF-DA in RAW 264.7 cells was confirmed by representative photographs obtained by means of fluorescence microscopy, either in the absence (Fig. 3B) or in the presence of DCF-induced fluorescence (Fig. 3C).

### Lipid peroxidation

As shown in Fig. 3D, the generation of thiobarbituric acid reactive substances (TBARS) increased significantly in A549 cells (*P* < 0.01) and, to a greater extent, in Raw 264.7 cells (*P* < 0.001) exposed to slag powders, irrespective of granulometry. An increased lipid peroxidation also occurred in the cells exposed to untreated asbestos. In A549 cells, generation of TBARS induced by asbestos was slightly but significantly higher than that induced by slag powders of any granulometry (*P* < 0.05).

### DNA damage

**Figure 4:**
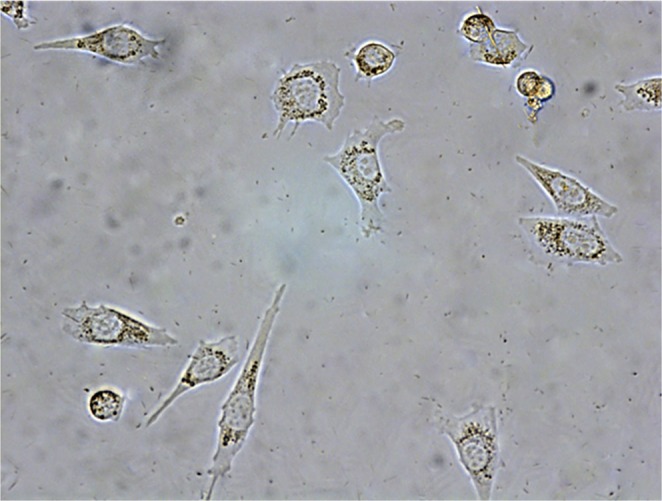
Microphotograph showing internalization of SHS- generated powder particles in A549 cells. Magnification: 360x.

**Figure 5:**
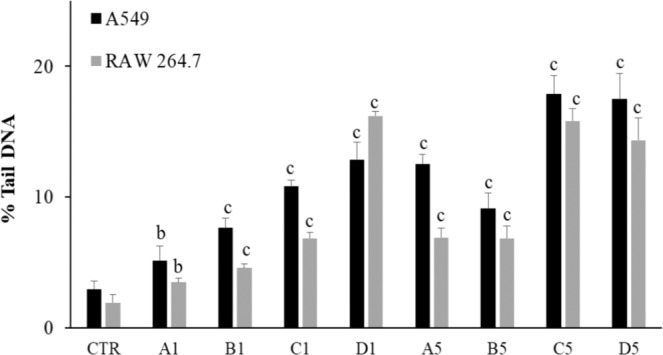
% Tail DNA, as evaluated by means of the Comet test, in A549 and Raw 267.4 cells after exposure for 12 h to the lowest (0.33 µg/ cm^2^) and to the highest concentrations (15.62 µg/cm^2^) either of slag powder particles or of asbestos. The columns report the means + SD of triplicate analyses. Statistical analysis: ^b^*P* < 0.01 and ^c^*P* < 0.001 *vs*. controls.

The photograph shown in Fig. 4 provide examples of internalization of SHS- generated powder particles in A549 cells. Figure 5 reports the percentage of DNA in the tail (TDNA %) as an indicator of DNA damage. The results obtained show a dose-related significant increase in DNA damage in both cell lines, in comparison with controls. A strong inverse correlation between DNA damage and particle diameter was observed. At the lower tested doses, the DNA damage caused by untreated asbestos was considerably higher than the one caused by slag powder particles, whereas at the highest tested doses the asbestos-related DNA damage was of the same order of magnitude as the one caused by the smallest slag powder particles.

### Chromosomal aberrations

Table 3 shows the frequency of micronucleated (MN) cells scored after treatment of either cell line with the different slag powder particles of varying granulometry (A–C). Asbestos (D) was used as a positive control. Asbestos treatment elicited a considerable and statistically significant increase in MN formation in both A549 and RAW 264.7 cells. Treatment with particles of the smallest diameter (<2 µm) increased the frequency of MN 1.8-fold in A549 cells and 1.9-fold in RAW 246.7 cells, but these differences did not prove to be statistically significant. The cytokinesis-block proliferation index (CBPI), which is an index of the cytotoxicity of test samples, was not affected by any treatment. The percentage of cytostatic cells in the variously treated samples considerably increased when the cell lines were inoculated with asbestos. A more moderate increase was also observed in RAW 246.7 cells exposed to slag powder particles of smallest granulometry. Thus, exposure to asbestos increased both the frequency of MN and the percentage of cytostasis in both A549 and RAW 264.7 cells.

**Table 3:** Frequency of micronuclei (MN), cytokinesis-block proliferation index (CBPI) and cytostasis (Cyt) in A549 and Raw 267.4 cell lines either untreated (Ctr) or exposed to asbestos (D) or to slags powder of varying granulometries (A < 10 µm, B < 5 µm, C < 2 µm).

Cell lines	Sample (15.62 µg/cm^2^)	MN/1000 cells	CBPI	Cyt %
A549 cells	Ctr	1.81	1.87 ± 0.15	—
	A	2.98	1.88 ± 0.08	1.15
	B	3.01	1.87 ± 0.07	0
	C	3.20	1.88 ± 0.13	1.15
	D	9.59^a^	2.03 ± 0.05	18.39
RAW 246.7	Ctr	0.82	1.64 ± 0.09	—
	A	0.84	1.65 ± 0.10	1.56
	B	0.88	1.66 ± 0.12	3.21
	C	1.60	1.71 ± 0.11	10.94
	D	7.60^a^	1.83 ± 0.05	29.69

Statistical analysis: ^a^*P* < 0.05 *vs*. control.

## Discussion

The results of the present study provide evidence that processing of wastes containing chrysotile fibres by SHS treatment alters their chemical composition and ultrastructural appearance. In parallel, there was an attenuation of oxidative alterations, which occurred in the absence of any lipid peroxidation alterations, as well as a decrease of the DNA damaging activity and especially of chromosomal aberrations. FUS-ICP analyses were consistent with the finding that the bulk composition of SHS-treated slags represents a mixture of SHS reagents (Mg and Al metals and Fe oxide) and reactant ACW, and the SEM images confirmed the pervasiveness of the SHS reaction and the absence of fibre remains.

Our *in vitro* study evaluated the effects of both untreated asbestos and slag powders in two cell lines that reproduce an alveolar environment. The results are consistent with the fact that asbestos fibres are able to generate widespread damage in the cellular environment. The biological activities of asbestos and its mutagenic action depend on several factors, including the size, shape, surface features and crystallinity of fibres, their chemical composition and physical durability, the route and duration of exposure, and the dose inhaled^17^. Asbestos fibres may induce mutagenicity by interfering with the process of cytokinesis through direct interaction with the mitotic spindle. Their indirect ability to produce reactive oxygen species (ROS) species may also induce genotoxicity^17^.

During breathing, particulate material and fibres that are not filtered out by the nose and bronchioles reach the alveolar space, where they may either be phagocytized by alveolar macrophages or penetrate the alveolar epithelium and be absorbed directly into the bloodstream^18^. As reported by Terzano^19^, ultrafine particles can inhibit phagocytosis in the lung, consequently generating an inflammatory response causing epithelial cell damage, and potentially facilitating entry into the interstitium. Specifically, asbestos fibres are known to generate an early pathogenic response involving cellular distress, DNA damage and alterations of the gene expression that governs proliferation, apoptosis and inflammation^20^.

The observed redox imbalance in cultured cells exposed either to asbestos or to small size powders reflects their ability to generate oxidative stress following contact with the cells of the epithelium and alveolar macrophages. More intense was the alteration of the redox status in RAW 264.7 cells exposed to untreated asbestos, which is likely to be attributed to the higher internalization of fibres by these monocyte/macrophage cells^21^. Oxidative stress is the central mechanism involved in the carcinogenicity induced by asbestos and may be responsible for deleterious effects in extra-pulmonary organs. Several studies on asbestos exposure have attributed the generation of ROS to Fenton-type reactions catalysed by iron on the surface of the fibre. ROS may be generated after phagocytosis of the fibre by alveolar macrophages or neutrophils^22^.

The results obtained show that the moderate ability of untreated asbestos to alter the redox status in pulmonary A549 cells was considerably lower in SHS-treated samples. Such a biological activity was lost in SHS-generated particles of large-medium granulometry, whereas it persisted when RAW 264.7 cells were exposed to small size particles, especially when challenged with the highest doses tested. This finding is consistent with the demonstration that environmental fine particulate matter is able to cross biological membranes in the same cells, where they induce lysosome activation^23^. For the same reason, the generation of lipid peroxides by untreated asbestos was considerably higher in RAW 264.7 cells than in A549 cells. Such a biological activity was unchanged when both types of cells were exposed to SHS-treated particle slags. Taking into account that the examined SHS products contained large amounts of iron, it is noteworthy that RAW 264.7 cells have been found to be susceptible to lipid peroxidation induced by FeSO_4_^24^.

In parallel, untreated chrisotyle fibres induced genomic alterations, which were of the same order of magnitude in RAW 264.7 cells and A549 cells, as detected both by comet assay and by cytokinesis-block MN test. The comet assay under alkaline conditions detects DNA strand breaks, both single-stranded and double-stranded, whereas the MN test detects chromosomal aberrations due either to chromosome breakage (clastogenic effect) or to loss of chromosomes (aneugenic effect)^25^. In particular, the cytokinesis-block MN test is a comprehensive system for measuring DNA damage, cytostasis and cytotoxicity. The DNA damage induced by asbestos may occur through two different mechanisms. The first one is damage to the nucleus due to penetration of cells by the smallest particles. The second mechanism involves generation of ROS, such as O_2_^−^ (superoxide radical), ^•^OH (hydroxyl radical) and H_2_O_2_ (hydrogen peroxide), probably due to the molecular oxygen reaction with structural metal species of the particles incorporated^26,27^ such as iron, which seems to play a role on the catalyst of redox-active reaction^28^. Irrespective of their size, the particles react with the cell membranes, which are the first target of free radicals. The generated ROS, which trigger formation of TBARS, have a limited range of action. DNA damage, consequent to the cellular redox state, is likely to be accentuated by the presence of smaller particles that are capable of reaching the nucleus more easily.

In contrast, no significant increase in chromosomal aberrations was observed in either pulmonary or monocyte/macrophage cell cultures exposed to SHS-treated chrysotile fibres of varying granulometry. However, the same material still caused DNA damage detectable by comet assay, presumably due to the high content in metals, such as iron and aluminium, which are able to promote a Fenton-like reaction *in vitro*, even more when they are combined^29^.

In conclusion, our results provide further evidence that asbestos fibres cause a redox status imbalance, lipid peroxidation, DNA damage in the form of single- and double-strand breaks, and chromosomal aberrations in cultured cells that mimic the alveolar environment. By contrast, when cultured cells were exposed to SHS-treated slag powders, a similar increase in oxidative stress ensued, but no chromosomal effect was observed. These results show that slag powders can cause some deleterious effects due to oxidative stress. It is however noteworthy that contact with slag powders did not significantly increase the number of micronuclei. This finding is probably the effect of the inertisation process, with loss of the asbestos fibrous structure following SHS reactions, as demonstrated by the analysis performed by means of SEM analyses.

## Materials and methods

### Cell cultures

To assess the biological effects of particles, both A549 cells and RAW 264.7 cells, obtained from ATCC (Rockville, MD, USA), were used. Both cell types were cultured and maintained in DMEM supplemented with 10% foetal calf serum (FCS), 100 IU ml^−1^ and penicillin/streptomycin (100 μg ml^−1^). The cells were grown in 75 cm^2^ culture flasks in a humidified 5% CO_2_ atmosphere at 37 °C and were supplied with fresh culture medium every 48 h. When 80–90% confluence was attained, both monolayers were subcultured and used for the experiments.

### Self-propagating high-temperature synthesis (SHS)

SHS exploits the heat produced by an alumino-thermic reaction, which is based on an oxidoreduction reaction between metallic oxide and aluminium or another reducing reagent (in our case, magnesium or aluminium)^30,31^. The reaction is associated with strong exothermicity, and proceeds in a self-sustained mode by processing continuous batches of mixed asbestos waste and oxide with an optimal feed rate that matches the reaction speed. The reaction occurs under Ar flow (Siad 99.998%). An oxy-acetylene torch triggers the reaction at the head of the pre-heated asbestos-containing waste (ACW batch), containing loose fibres and asbestos cement. The samples to be tested in the cell cultures were prepared by dispersing the dust with different sizes in deionized water through sonication, with a final concentration of the stock batch of 10 mg/ml.

### Chemical analysis of SHS-treated slags

The optical analyses were carried out by a digital portable microscope (Dinolite AM-7013MZT, Dino-Lite Europe IDCP B.V., Naarden, The Netherlands) under 50× to 200× magnification. The X-ray diffraction patterns of samples were performed on a Philips PW 1140 diffractometer using the Bragg– Brentano geometry, with Cu K radiation (λ = 0.15406 nm), input 40 KV; electric current intensity: 20 mA; start angle 3°; total scanning: 80 °C.

### SEM analyses

SEM analyses were carried out by using a Tescan Vega 3 LM scanning electron microscope equipped with an Apollo X detector and a Microanalysis TEAM Energy Dispersive Spectrometry (SEM/EDS: TESCAN, Brno, Czech Republic). The samples were metalized with a 25–50 nm layer of Au by cathodic sputtering (Quorum Q150T ES). Microphotographs were acquired with an optimized ratio between back scattered and secondary electrons in order to preserve chemical and morphologic information.

The starting materials for the bioassays were SHS slags that had been hand-ground and sieved to separate the fractions, with grain sizes of <10 μm, <3 μm and <2 μm, referred to as samples A, B, and C, respectively. A specimen of pure chrysotile, previously characterised^13^, was named sample D. The bulk composition of 5 slags was analysed by LiB Fusion followed by Inductively Coupled Plasma Mass Spectrometry (ICP MS) analysis.

### Cell viability assay

To evaluate the toxicity of the powders obtained by grinding, a crystal violet assay was performed in A549 and Raw 264.7 cells. A threshold cell death higher than 30% was assumed to indicate a toxic effect. Exponentially growing cells were seeded for culture in 96-well plates (4 × 10^4^ cells/well) and incubated in complete medium. After 24 h, the medium was replaced with different dilutions of samples in fresh medium (100 μL) containing 2% FCS, using 8 wells per dose. The cells were inoculated with 0.33, 1.00, 3.33, 7.58, and 15.62 µg/cm^2^ of each sample. These doses were suggested in previous studies^32,33^. After incubation for 24, 48, and 72 h, the monolayers of cells were rinsed three times with phosphate buffered saline (PBS), fixed with 2.5% glutaraldehyde for 10 min, stained with a 1% crystal violet solution, and dried. Subsequently the cells were solubilized in a methanol/acetone (4:1, v/v) solution (150 μL/well) and the different colour intensity was measured by means of a microtitre plate reader (Tecan Italia, Cernusco sul Naviglio, Milan, Italy). The value reported from each replicate was converted to a percentage and compared with the untreated control value (100%).

Simultaneously, in order to investigate the possible toxic effect of salt residues in the particle samples, dialysis through a semi-permeable cellulose acetate membrane was performed overnight in deionized water. The washed samples obtained were tested with crystal violet assay, as reported above. None of the dialysed samples differed significantly from the non-dialysed samples (data not shown). Therefore, in the following tests, dialysis was not performed.

### Redox status

The cellular redox status was evaluated by means of the molecule 2′,7′-dichlorofluoresceindiacetate (DCF-DA). This non-fluorescent compound easily crosses cell membranes and quickly responds to alterations in intracellular iron signalling or enhanced peroxidase activity^34^. A549 and RAW 264.7 cells (4 × 10^3^ per well) were seeded in 96-well plates. After 24 h, the cells were washed and loaded with 2 μM of DCF-DA in DMEM medium containing 2% FCS, and maintained at 37 °C for 30 min. Subsequently, the cells were washed twice and treated either with different concentrations (0.33, 1.00, 3.33, 7.58, and 15.62 and µg/cm^2^) of slag powders at different granulometries or with untreated asbestos, using 8 replicates per dose. After incubation for 3 h at 37 °C, fluorescence analysis was carried out by means of a Perkin-Elmer LS3B spectrophotometer. The fluorescence in each well was recorded at 495 nm *E*_*x*_ and 530 nm *E*_*m*_, and the change in redox status was expressed as the intensity of fluorescence per number of cells, counted after crystal violet staining.

### Lipid peroxidation products

Lipid peroxidation was assessed by measuring the generation of thiobarbituric acid reactive substances (TBARS). A549 and RAW 264.7 cells were exposed for 12 h to the highest concentration (15.62 µg/cm^2^) either of slag powders of different granulometries or with untreated asbestos (D). Briefly, the cells were mixed with sodium dodecyl sulfate, acetate buffer (pH 3.5), and aqueous solution of thiobarbituric acid. After heating at 95 °C for 60 min, the red pigment produced was extracted with n-butanol-pyridine mixture and estimated by the absorbance at 532 nm^35^. Malondialdehyde (MDA) was used as a standard The results are expressed as nmol of MDA equivalent per mg of protein.

### Single-cell gel electrophoresis (SCGE)

The alkaline comet assay, or SCGE, was performed according to Tice *et al*.^36^, with some modifications. Briefly, A549 and RAW 264.7 cells were seeded in 6-well plates and underwent treatment in duplicate for 12 h with the lowest (0.33 µg/cm^2^) and highest concentrations of particles (15.62 µg/cm^2^) in order to detect a potential genotoxic damage response. After incubation, the cells were detached by means of 0.25% trypsin and 1 mM EDTA. Viability of cells was checked by trypan blue exclusion^37^. A viability of at least 80% was a prerequisite to proceed. Thereafter, an aliquot of cells (~20,000) was embedded in 75 μL of 0.5% low melting-point agarose and layered onto slides, which were immediately covered with a coverslip and allowed to solidify at 4 °C.

The procedure was carried out according to a previously described method^38^. After removal of the coverslip, a second layer of low melting-point agarose was added to the slide, which was then immersed in cold lysing solution (2.5 M NaCl, 100 mM ethylenediaminetetraacetic acid, 10 mM Tris, pH 10, 1% Triton X-100, and 10% dimethyl sulfoxide) for 1 h at 4 °C. Subsequently, the samples were rinsed in alkaline solution (0.3 M NaOH, 1 mM ethylenediaminetetraacetic acid, pH 13) and underwent electrophoresis, which was carried out in fresh alkaline solution for 30 min at 25 V (0.66 V/cm), adjusted to 300 mA. After a neutralisation step (0.4 M Tris-HCl, pH 7.5), the acquisitions of nuclei imbibed in each slide and stained with ethidium bromide (2 μg/mL) were obtained by means of a fluorescence microscope and a digital camera at a 200× magnification. Images were acquired of 100 randomly selected nuclei per sample, and the analysis was conducted by means of CASP (Comet assay software project, http://www.casp.sourceforge.net). The results were expressed in terms of DNA percentage in the tail (TDNA %).

### Evaluation of micronucleated cells

In order to assess the genotoxic effect of particles in RAW 264.7 and A549 cells, the MN assay was performed as follows. Briefly, we seeded about 20 × 10^4^ cells in each well of a 6-well plate, and we prepared two paired cultures for each size of particle. After 24 h, the cultured cells were inoculated with particles of three different size. Untreated asbestos was used as a positive control. We then added cytochalasin B (4 µg/mL), 48 h after preparation of the cultures, in order to block the cytokinesis of mitotic cells^39^. After 28 h, the cells were harvested, treated with a 0.075 M KCl hypotonic solution for 2 min, prefixed in 3:5 methanol/acetic acid, and washed twice with a 6:1 methanol/acetic acid fixative solution.

Two microscopic slides were obtained by smearing each sample. The slides underwent mild acid hydrolysis in a 5 N solution of HCl for 1 h. The slides were then rinsed in distilled water and stained with Schiff’s reagent (Sigma Chemical Co., St. Louis, MO) for 30 min, washed thoroughly in distilled water, and left for 5 min under running tap water in order to intensify the pink staining from the Schiff’s reagent. Finally, the slides were counterstained with 1% light green for 35 min, washed in tap water, blotted dry, and mounted. One thousand cells from each individual culture replicate were examined as blind samples for the presence of micronucleated cells under an optical microscope at 1000× magnification. The cytokinetic activity of the cells was also assessed on the basis of the cytokinesis-block proliferation index (CBPI), which was calculated by means of the formula^40^:

$${\rm{CBPI}}=\frac{{\rm{No}}.\,{\rm{mononucleate}}\,{\rm{cells}}+2\,{\rm{x}}\,{\rm{No}}.\,{\rm{binucleate}}\,{\rm{cells}}+3\,{\rm{x}}\,{\rm{No}}.\,{\rm{multinucleate}}\,{\rm{cells}}}{{\rm{Total}}\,{\rm{number}}\,{\rm{of}}\,{\rm{cells}}}$$

The CBPI indicates the number of cell cycles per cell during exposure to cytochalasin B in relation to the number of nuclei in treated cultures. The cytostatic effect was in turn calculated from the measured CBPI values as follows:

Cytostasis % = {100 − 100 × [(CBPI exposed cells - 1)/(CBPI control cells - 1)]}

### Statistical analyses

The analyses were performed by means of STAT View software. The results regarding cell viability, redox status and lipid peroxidation were expressed as means ± SD of multiple individual experiments, and data were analysed by means of one-way analysis of variance (ANOVA) with *post hoc* testing using Dunnett’s multiple comparison test. A *P*-value of <0.05 was considered as statistically significant. Fisher’s exact test was performed in order to determine any statistically significant difference between each treatment and the control with regard to the frequency of micronucleated cells.
